# Transcriptomic, proteomic and metabolomic analysis of UV-B signaling in maize

**DOI:** 10.1186/1471-2164-12-321

**Published:** 2011-06-16

**Authors:** Paula Casati, Mabel Campi, Darren J Morrow, John F Fernandes, Virginia Walbot

**Affiliations:** 1 Centro de Estudios Fotosintéticos y Bioquímicos (CEFOBI), Facultad de Ciencias Bioquímicas y Farmacéuticas, Universidad Nacional de Rosario, Suipacha 531, 2000 Rosario, Argentina; 2 Department of Biology, 385 Serra Mall, Stanford University, Stanford, CA 94305-5020, USA

## Abstract

**Background:**

Under normal solar fluence, UV-B damages macromolecules, but it also elicits physiological acclimation and developmental changes in plants. Excess UV-B decreases crop yield. Using a treatment twice solar fluence, we focus on discovering signals produced in UV-B-irradiated maize leaves that translate to systemic changes in shielded leaves and immature ears.

**Results:**

Using transcriptome and proteomic profiling, we tracked the kinetics of transcript and protein alterations in exposed and shielded organs over 6 h. In parallel, metabolic profiling identified candidate signaling molecules based on rapid increase in irradiated leaves and increased levels in shielded organs; pathways associated with the synthesis, sequestration, or degradation of some of these potential signal molecules were UV-B-responsive. Exposure of just the top leaf substantially alters the transcriptomes of both irradiated and shielded organs, with greater changes as additional leaves are irradiated. Some phenylpropanoid pathway genes are expressed only in irradiated leaves, reflected in accumulation of pathway sunscreen molecules. Most protein changes detected occur quickly: approximately 92% of the proteins in leaves and 73% in immature ears changed after 4 h UV-B were altered by a 1 h UV-B treatment.

**Conclusions:**

There were significant transcriptome, proteomic, and metabolomic changes under all conditions studied in both shielded and irradiated organs. A dramatic decrease in transcript diversity in irradiated and shielded leaves occurs between 0 h and 1 h, demonstrating the susceptibility of plants to short term UV-B spikes as during ozone depletion. Immature maize ears are highly responsive to canopy leaf exposure to UV-B.

## Background

Under normal solar fluence, UV-B damage to macromolecules is balanced by their subsequent repair or replacement. Sporadic ozone depletion results in local "ozone holes" and spikes in terrestrial UV-B exposure. These periodic, but unpredictable UV-B spikes increase intensity up to 10-fold in both the polar and temperate zones [1]. Furthermore, the ozone shield against UV-B is not expected to stabilize at 1950 levels until ~2050 [2]; consequently, determining the molecular bases for acclimation to normal fluence and tolerance of higher fluence UV-B are important factors in sustaining crop yield as the world's population continues to increase.

Previously, we established that maize lines have different UV-B tolerance, primarily because higher flavonoid sunscreens are correlated with fewer stress responses [3,4]. Additionally, high altitude (> 2000 m) landraces naturally exposed to greater UV-B exhibit higher UV-B tolerance because they have both higher flavonoids and greater chromatin remodeling capacity [4,5]. Conversely, temperate maize with knockdowns in chromatin remodeling factors exhibit adult tissue hypersensitivity and seedling lethality after mild UV-B supplementation [5,6]. These and studies on other plants implicate both metabolite and gene expression responses as critical for short-term acclimation to UV-B and as examples of plant adaptation to this environmental variable [7-9].

In a pilot experiment we discovered that shielded organs, such as leaves wrapped in UV-B filters and immature ears encased in the husk leaves, show transcriptome changes within an hour or two after canopy leaves receive UV-B [10]. Because such systemic responses can impact yield by modulating ear or kernel growth, identifying signals produced in sunlit leaves that alter reproductive organs should elucidate how UV-B decreases plant yield beyond what is predicted from the modest impact of UV on photosynthesis. Now we report a 1 to 6 hour time course of transcriptome and proteome responses in irradiated leaves, shielded leaves, and immature ears to unravel the systemic physiological and developmental responses in exposed and shielded organs. In parallel, metabolic profiling was used to search for candidate signaling molecules by charting increases in irradiated and then shielded organs. Integrating the datasets, we determined whether the biosynthetic, sequestration, or degradative pathways for candidate signaling molecules are regulated, at least in part, by UV-B exposure effects on transcript or protein abundance levels.

## Results

### Microarray hybridization design and reliability

**Figure 1:**
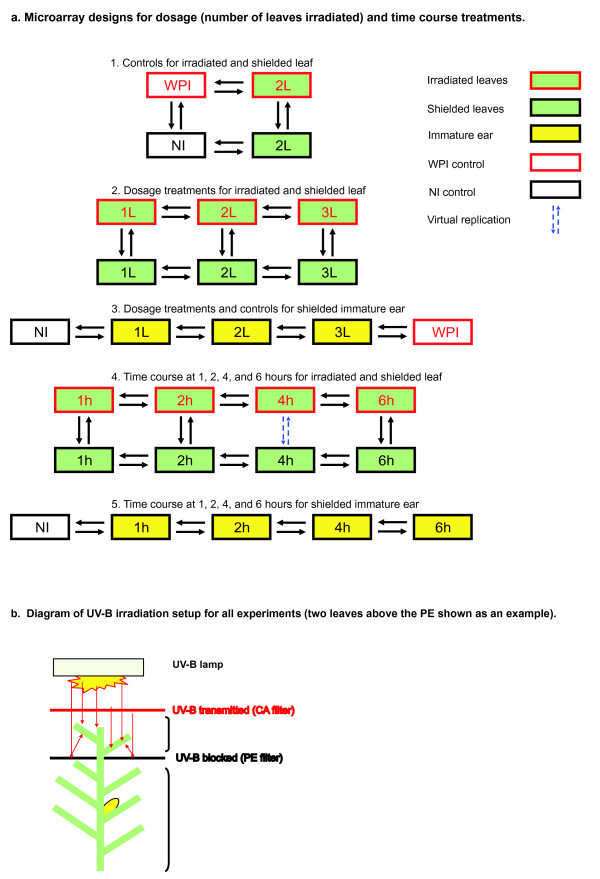
**Microarray design and UV-B irradiation apparatus**. (a) Microarray hybridization design with direct comparisons to measure UV-B effects on signaling initiation from irradiated to shielded tissues: 1) controls, 2) dosage comparison for leaves, 3) dosage comparison for ears, 4) time course comparison for leaves, and 5) time course comparison for ears. The dosage treatments were all done at a standard time of 4 h irradiation, and all time course treatments had two leaves exposed above the PE. Each arrow represents a pair of samples hybridized on one array; in every case there is one cy3-labeled sample compared to one cy5-labeled sample. For each sample type there were four biological replicates, generating at least four hybridization comparisons, except where noted. Dotted blue arrows signify a "virtual" replication in which the comparison of the same samples occurred elsewhere in the design. (b) UV-B-irradiation apparatus for selective leaf irradiation. Irradiated leaves were threaded through slits in the PE plastic to permit UV-B irradiation and collected after irradiation; mature shielded leaf samples were collected immediately below the cut-off filter. Immature ears were dissected from encasing husks leaves after treatments. For the control plants, the PE filter plastic was removed from the apparatus. No UV-B irradiation (NI) control plants were sampled without moving under the apparatus and thus did not receive UV-B; whole irradiated plants (WPI) received UV-B without any PE shielding. See Materials and methods for details of the tissue sampling protocol.

Two types of comparisons were performed: dosage treatments based on the number of leaves irradiated and time course treatments from 0 to 6 hours. The hybridization schema is diagrammed in Figure 1a. Whole plant irradiation (WPI) and non-irradiated plants (NI) served as full UV-B and no UV-B controls. Experimental samples were recovered from plants with partial shielding (Figure 1b); top canopy leaves were irradiated but other fully expanded leaves and immature ears were shielded from direct UV-B exposure. Sensitive, custom-designed Agilent^® ^4 × 44 K arrays with 60-mer probes and internal spike-in controls were used to quantify transcript abundance for ~39,000 genes. Four independent biological replicates of each sample type, created by pooling samples from 4 individual plants, were used to assess the transcriptome differences. Symmetrical dye labeling minimized systematic errors [11] using criteria described in Materials and methods. The correlation in quantitative comparisons among biological replicates was r^2 ^= 0.90 - 0.99 (data not shown).

### Transcriptome analysis of leaves from UV-B-irradiated and non-irradiated plants

**Figure 2:**
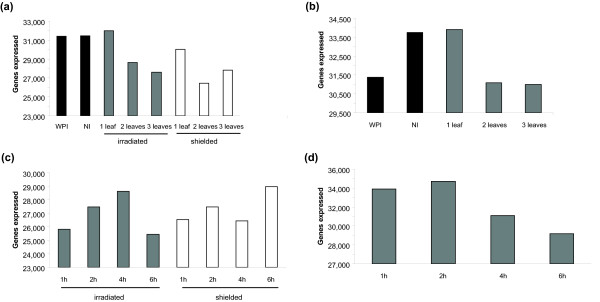
**Transcriptome size in samples varying the canopy exposed and after different UV-B duration**. (a and b) Transcriptome size in (a) whole irradiated (WPI), and non-irradiated plants (NI), and irradiated and shielded leaves from 1, 2 and 3 CLI plants, and (b) ears from CLI plants after 4 h of irradiation. (c and d) Transcriptome size in (c) irradiated and shielded leaves from 1, 2 and 3 CLI, and (d) immature ears from plants with two irradiated leaves for 1, 2, 4, and 6 h of UV-B.

We first compared the transcriptome of the topmost leaves from plants exposed to 4 h UV-B (whole plant irradiation, WPI) to that from non-irradiated plants (NI): 203 transcripts decreased while 213 increased at least 2-fold (p < 0.05). These 416 transcripts represent ~2% of the leaf transcriptome (Figure 2a and 2b). At a 1.5-fold cut-off 714 transcripts are down-regulated and 862 up-regulated, summing to ~4% of the transcriptome.

Figure S1 in Additional file 1 shows the GO classification of these transcripts. Genes regulated at least 2-fold (Table S1 in Additional file 2) include 6 transcripts encoding enzymes in the flavonoid pathway and 3 transcripts for enzymes in DNA repair, confirming processes often uncovered during plant acclimation to UV-B. A myo-inositol-1-phosphate synthase transcript is down-regulated by UV-B as reported previously [3,10]. Using a 1.5-fold cutoff, more gene classes are represented (Table S1 in Additional file 2): up-regulated types include ribosomal proteins and translation factors, enzymes for detoxification of reactive oxygen species, chromatin remodeling proteins, and transcription factors (TFs). Among the down-regulated types are photosynthetic enzymes, TFs, and enzymes in starch biosynthesis. The repression and induction of TFs as well as the specific metabolic processes altered by UV-B confirm previous reports [3,6,10].

### What proportion of the plant canopy must receive UV-B to alter transcripts in shielded organs?

Canopy leaf irradiation (CLI) plants were screened by polyester (PE) that absorbs UV-B with the first, top two, or top three leaves threaded through the filter to receive UV-B-irradiation from lamps covered with cellulose acetate (CA) to filter radiation < 290 nm (Figure 1). One leaf exposure caused a small net increase of 513 transcripts versus the NI control with a slightly higher number of transcripts turned on (1397) versus off (884), plus an additional 1181 transcripts were either up- or down-regulated. In contrast, two and three leaf exposures resulted in a decrease of over 2500 transcripts and 3400 transcripts, respectively, while the total up- and down-regulated genes approximately doubled with each additional exposed leaf (2097 and 4039). Two or three exposed leaves similarly impacted shielded tissues (Figure 2a and 2b). In a time course, total transcript diversity in irradiated leaves decreased by > 5000 transcript types in 1 h compared to the no-UV-B (NI) control leaves, increased from 1 to 4 h, and then declined sharply at 6 h (Figure 2a and 2c), reinforcing previous observations that distinctive responses occur after 4 h [12]. In shielded leaves, transcript diversity dropped by 4000 transcript types in 1 h and then increased over the time course to restore 94% of transcript diversity at 6 h. Ears have higher transcriptome complexity than leaves (Figure 2b) and sustain this through 1 and 2 h exposures; at 4 h, two and three leaf CLI decreases transcript diversity similarly (~2000) and then transcript diversity is decreased further from 4 to 6 h (Figure 2d). In summary, transcript diversity decreases > 10% in irradiated and shielded tissues, and each tissue type is distinctive over a 1 to 6 h time course.

**Figure 3:**
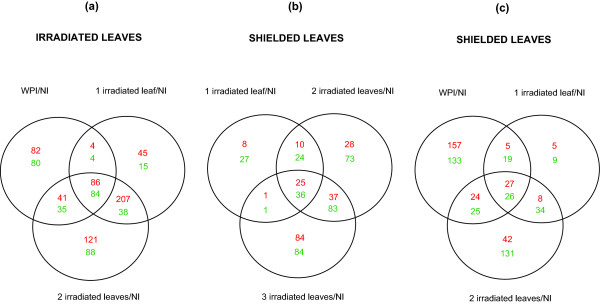
**Transcriptome changes in irradiated and shielded leaves from plants with different canopy leaf exposure**. Up-regulated genes are red, down-regulated genes are green. (a) Intersection of genes differentially expressed in irradiated leaves. (b and c) Intersection of genes differentially expressed in shielded leaves. Each sample was compared to plants under control conditions with no UV-B (NI).

The datasets for 4 h WPI and CLI were evaluated for common responses (Figure 3a; Tables S2 and S3 in Additional file 2). It is striking that more transcriptome changes are found in CLI than in WPI leaves: 483 transcript alterations with one leaf irradiated and 700 for two leaves. A subset of these may be responses to minor damage from threading leaves through the PE plastic ~24 h before treatments. Of the 416 transcripts differentially expressed more than 2-fold in WPI leaves compared to non-irradiated plants, only 162 (82 up and 80 down) are unique to this treatment (Table S2 in Additional file 2). Regulation of these 162 transcripts requires either that the entire plant receives the stimulus or that organs in addition to top irradiated leaves must sense UV-B directly. After assignment of GO terms (Additional file 1), up-regulated transcripts include transporters, kinases that participate in signaling, proteinases, and TFs. Exemplar down-regulated genes include those encoding heat shock proteins (HSPs), protein kinases, and other transporters.

In Figure 3a comparing all 3 CLI treatments with a 2-fold cutoff, 41% of transcripts (170 of 416) are commonly UV-B-regulated. Within these, all 6 genes in the flavonoid pathway (Table S3 in Additional file 2) and a transcript for a putative DNA repair enzyme, RadA-like protein, are up-regulated. We infer that mechanisms for avoidance and repair of UV-B damage act within exposed leaves irrespective of the irradiated area. In addition, two cysteine proteases and 17 TFs are induced after WPI and all CLI treatments (Table S4 in Additional file 2). As in the WPI results, the myo-inositol-1-phosphate synthase transcript shows a decreased level for all three CLI treatments; myo-inositol-1-phosphate may play a role in programmed cell death in Arabidopsis, however, in UV-B-treated maize it may be part of a metabolite signaling pathway [13]. Eight HSPs are down-regulated in all three CLI treatments (Table S3 in Additional file 2).

Two transcripts encoding maize homologues of UVR8, a UV-B-specific signaling component that orchestrates expression of vital UV-protective functions in Arabidopsis [9,14], are down-regulated in all irradiated maize leaves. Although UVR8 has not been reported to be UV-B-regulated, our inspection of Arabidopsis microarray data with Genevestigator^© ^uncovered down-regulation of UVR8 by UV-B (data not shown).

### Transcriptome analysis of shielded leaves from UV-B-irradiated plants

PE-covered leaves and immature ears should respond to UV-B radiation only if exposed leaves transmit a signal(s). Shielded leaf samples were collected from the two leaves immediately below the PE filter after 4 h irradiation. We consider the shielded leaves of these mature plants (leaf expansion completed) to be physiologically similar to irradiated leaves. As shown in Figure 3b, at the 2-fold cutoff, shielded leaves from single leaf CLI plants show the fewest (132) transcript changes, with 73% of them shared with the 2 and 3 leaf treatments. Sixty one transcript alterations (12% of the total) are shared by all shielded leaf samples and represent core responses triggered by UV-B perception. Among these, 25 are increased including 4 DNA binding proteins of which at least two are TFs (Table S4 in Additional file 2). Interestingly, a serine:threonine kinase and a cysteine protease are also increased in both irradiated and shielded samples (Tables S1 and S4 in Additional file 2). UVR8 and myo-inositol-1-phosphate synthase are among the 36 down-regulated transcripts (Table S4 in Additional file 2) shared with directly irradiated leaves. Although a number of HSPs have been reported to be up-regulated by UV-B [3], a decrease in the levels of HSPs 82, 101 and clpB have been reported in the W23 maize inbred [6], and here we find two HSPs decreased in shielded organs (Table S4 in Additional file 2). Using a 1.5-fold cut-off, 120 transcripts levels were altered in shielded leaves from 4 h single leaf CLI treatments, increasing slightly to 134 for 2 leaves (Table S4 in Additional file 2). Using this lower cut-off, genes for stress responses, a hypersensitive-induced response protein, and some TFs are increased. Down-regulated genes include additional HSPs, nitrate transporters, and TFs (Table S4 in Additional file 2). Of the 416 transcripts changed more than 2-fold in WPI plants, 53 were also differentially expressed in shielded leaves on 1 or 2 leaf CLI plants (Figure 3c, center element).

In summary, after 4 h UV-B exposure of canopy leaves, shielded leaves have 521 modulated transcripts. A subset of altered transcripts is shared with WPI leaves. We hypothesize that signals from currently UV-B-irradiated leaves are transmitted to currently shielded leaves where some acclimation factors are regulated similarly to irradiated leaves. These triggered responses may contribute to leaf acclimation when sunlight reaches currently shaded leaves.

### Analysis of transcriptome responses in shielded ears

**Figure 4:**
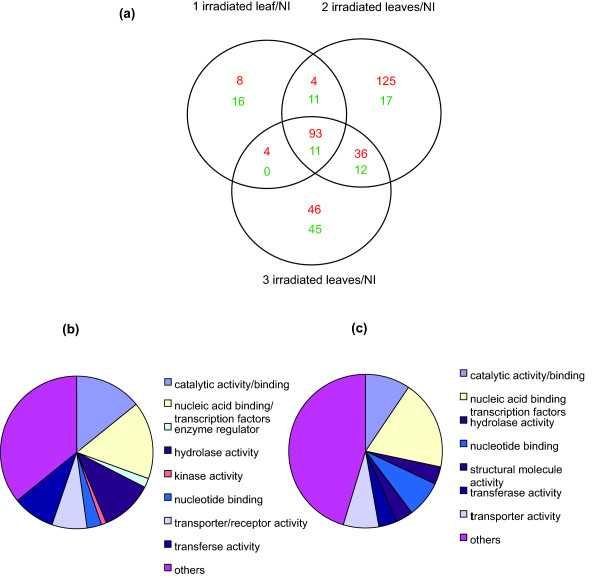
**Analysis of immature ear transcriptome changes**. (a) Transcriptome changes with varying canopy exposure. Each sample was compared to non-irradiated control plants (NI). Up-regulated genes are red, down-regulated genes are green. (b and c) GO classification of UV-B-regulated transcripts based on their putative function: (b) up-regulated and (c) down-regulated.

To better understand the impact of UV-B in non-irradiated tissues, we examined responses in immature 1-5 cm ears encased in husk leaves. Despite this protection, 34 genes were down-regulated by UV-B, while 8 were up-regulated in ears in a pilot study [10]. In the current experiments, we compared transcriptomes for WPI treatments, non-irradiated controls, and CLI treatments. Figure 4a shows that 428 transcripts are changed in one or more treatments. In all CLI treatments, 104 transcripts are altered at least 2-fold, and 243 are regulated at least 1.5-fold (p < 0.05, Table S5 in Additional file 2). The latter 243 transcripts were classified by GO categories (Figures 4b and 4c). In the up-regulated group, the major category is nucleic acid binding proteins, which includes TFs (16%, Figure 4b; Table S5 in Additional file 2). Thus, UV-B signaling from irradiated leaves to shielded immature ears induces TFs that likely modulate expression changes in other genes in response to UV-B. Another important group is represented by hydrolases (11%): proteinases, lipases, cell wall degradation, and pathogen response enzymes such as beta-1,3-glucanase (Table S5 in Additional file 2). Such hydrolases are commonly induced under other stress conditions [15-18], suggesting that some components of UV-B-induced systemic signaling elicit general stress responses in shielded organs. Transcripts for transport processes and receptors (7.4%) are an interesting category; examples in this group are a glycerol-3-phosphate transporter, aphid transmission P5, a MDR-like ABC transporter, a NOD26-like membrane integral protein ZmNIP1-1, and a peptide transporter-like protein (Table S5 in Additional file 2); these are candidates for transporting a signaling molecule from irradiated tissues into the cells of a shielded ear. The major group of down-regulated transcripts is also the nucleic acid binding group (18%, Figure 4c), confirming there is both induction and repression of TFs to modulate UV-B responses. As described for shielded leaves, there is also down-regulation of several HSP genes (Table S5 in Additional file 2), which we now classify as a general UV-B response in shielded tissues.

Despite the parallels in affected processes, few transcripts were shared between immature ears and shielded leaves from the 2-leaf CLI treatment: 9 transcripts at a 2-fold cutoff (1.5% of total regulated transcripts in both conditions) and 52 transcripts (2.4%) at a 1.5-fold cutoff (Figure S2 in Additional file 3 and Table S6 in Additional file 2). These low values are within the false discovery range in assigning expressed transcripts, and we conclude that shielded ears and leaves express distinctive gene suites after CLI.

### Time course of transcriptome: differential expression responses

**Figure 5:**
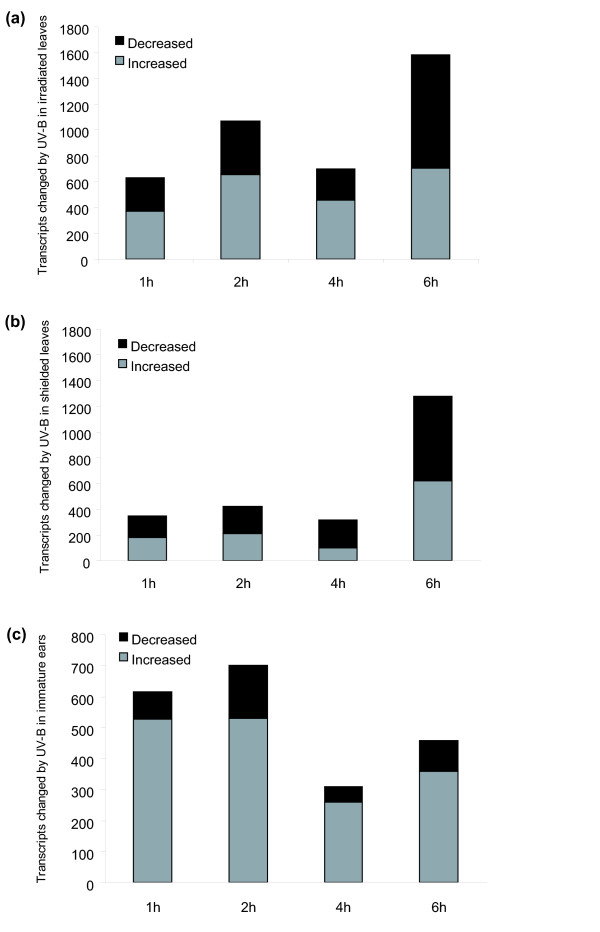
**Transcript changes in plants with 2 leaves exposed over 1, 2, 4, or 6 h**. RNA from (a) irradiated leaves, (b) shielded leaves, and (c) immature ears was used for the microarray experiments.

Figure 5a shows 628 transcripts that are changed by at least 2-fold (p < 0.05) in irradiated leaves after 1 h of UV-B compared to non-irradiated plants, increasing to 1073 transcripts after 2 h. Of the 628 transcripts, 76% also show altered expression after 2 h of UV-B (central element, Figure S3a in Additional file 4) whereas 153 transcripts are unique to 1 h UV-B and are likely involved in the earliest responses. Of the 66 uniquely up-regulated early transcripts (Figure S3a in Additional file 4), there are at least 8 TFs and 12 transcripts encoding signal transduction proteins (Table S7 in Additional file 2), that probably participate in early UV-B signaling. For example, a protein similar to Arabidopsis phytochrome and flowering time 1 protein (PFT1), a subunit of the Mediator complex [19], is an early response.

In shielded leaves, the total number of transcripts changed after 1 h of UV-B was lower than in irradiated leaves (348 transcripts, Figure 5b); 270 of these changes persist at 2 h (central element, Figure S3b in Additional file 4). The 78 uniquely modulated after 1 h (Figure S3b in Additional file 4) are candidate components of initial UV-B signaling in shielded tissues; this group includes transcripts for signal transduction proteins, TFs, and transporters (Table S7 in Additional file 2). A UVR8 homolog is up-regulated in shielded leaves after 1 h of UV-B but not at longer UV-B exposures nor in any irradiated samples (Table S7 in Additional file 2). This transcript may be induced at earlier times in irradiated leaves as an initial response to UV-B and then in shielded leaves with a time delay after transmission of an inducing signal. Thus, the down-regulation of UVR8 measured at longer UV-B exposures (Table S7 in Additional file 2) may be a feedback response to the treatment. Comparing 1 h irradiated versus shielded leaves, 106 transcripts are changed in a similar way at least 2-fold (p < 0.05) in both conditions: 56 are increased, including transcripts for signaling proteins and TFs, and 50 decreased (Table S7 in Additional file 2). A wound and phytochrome signaling involved receptor-like kinase is increased in both irradiated and shielded leaves after 1 h, emphasizing its potential as a general participant in UV-B signaling.

After 6 h of UV-B, more transcripts increased significantly in both irradiated (1584, 2-fold, p < 0.05) and shielded (1280, 2-fold, p < 0.05) leaves. This is a 2.5-fold increase with respect to the 1 h treatment for irradiated and a 3.7-fold increase for shielded leaves (Figure 5a and 5b). Fewer than 20% of these transcripts are shared with the 4 h treatment in irradiated (19%) and shielded (17%) leaves (central element, Figure S3a and b in Additional file 4). This observation reinforces the distinction between initial (up to 4 h) and later responses of 6 h or longer [12]. Genes in this category are described in Table S7 in Additional file 2.

Examining the time course for immature ears (Figure 5c) we find fewer total transcripts changed 2-fold (p < 0.05) by UV-B than in leaves; ears retain transcript diversity at 1 and 2 h because there are more up- than down-regulated genes. In ears, 82% of altered transcripts at 1 h (500) are also changed after 2 h of UV-B (Figure S3c in Additional file 4). Similar to shielded leaves, the 1 h CLI treatment induced transporters, TFs and signaling proteins (Table S8 in Additional file 2). Surprisingly, transcripts for multiple HSPs are transiently induced, and then down-regulated as a class after 4 h UV-B (Table S8 in Additional file 2); thus, as suggested for UVR8 in shielded leaves, the down-regulation observed at longer exposure times may be part of a feedback response to suppress physiological changes induced by initial UV-B perception.

Some early-induced transcripts remain differentially regulated throughout the time course: 82 in irradiated leaves, 19 in shielded leaves, and 103 in immature ears (Tables S7 and S8 in Additional file 2); these probably correspond to transcripts encoding proteins necessary for stress acclimation. Up-regulated examples for irradiated leaves include three phenylpropanoid pathway genes, a peroxidase PXC2, TFs of the WRKY type, and a PR protein (Table S7 in Additional file 2). In immature ears, 86 transcripts are induced > 2-fold at all time points (p < 0.05), including two phenylalanine ammonia-lyases, a peroxidase 13, and GSTs 20 and 22. Also up-regulated are transcripts encoding enzymes in cell wall degradation (Table S8 in Additional file 2). The up-regulation of these genes is specific for immature ears; it is possible UV-B induces some specific cell wall reorganization in these organs.

The impact of UV-B on fourteen major cellular processes was assessed by GO classification of transcripts from four expression types (those that were turned on or off or that were up- or down-regulated versus the NI control) for each of the 4 time points in the 6 h time course experiment (Figure S4 in Additional file 5). UV-B perception and systemic signaling to shielded organs has a major impact in all fourteen GO categories. The proportion of genes in each of the four expression types is distinctive for irradiated leaf, shielded leaf, and immature ears. For each of the fourteen categories and for each of the 4 time points, responses in the two types of leaf samples share more similarities than either does with the immature ear.

### Identification of UV-B induced metabolomic changes

As a first step in identifying potential signal molecules moving from irradiated leaves to shielded organs, we conducted metabolic profiling using GC-MS to find metabolites altered by a 4 h UV-B treatment. We identified 84 compounds, 30 of which had a statistically significant change (Figure S5 in Additional file 6, one way ANOVA). Levels of six metabolites were only increased under WPI treatment (valine, serine, asparagine, glycerate, hexa/heptadecanoic acid and an unidentified metabolite, Additional file 6). Levels of thirteen metabolites were changed by UV-B in both WPI and CLI treatments, whereas altered levels of the following were restricted to irradiated leaves: alanine, fructose, galactose, glucose, glucaric/galactaric acid, xylose, dopamine, dihydroascorbic acid dimer, 2-ketoglutaric acid, and three compounds in the phenylpropanoid pathway (shikimic acid, quinic acid, and trans-caffeoylquinic acid) (Additional file 6). All six UV-B-regulated genes in the flavonoid pathway show increased levels exclusively in directly exposed leaves (Tables S3 and S4 in Additional file 2), suggesting that these metabolites are not translocated to shielded tissues nor do mobile signals induce them in shielded organs.

**Figure 6:**
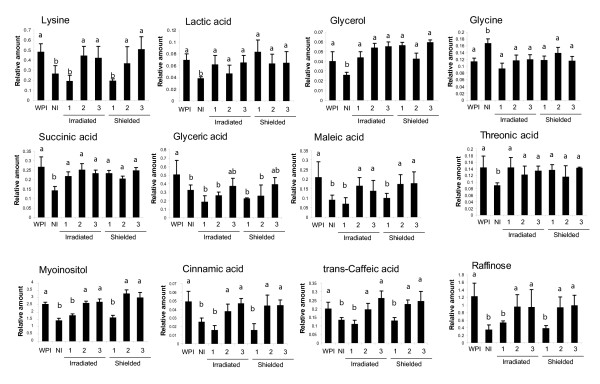
**Metabolic profiling of irradiated and shielded leaves from plants with varying canopy exposure to UV-B**. Metabolites changed in WPI, NI and both irradiated and shielded leaves from 1, 2 and 3 CLI plants are shown. Plants were irradiated during 4 h. For statistical analysis see Materials and methods.

Twelve metabolites were changed by UV-B in both irradiated and shielded leaves; some requiring a single irradiated leaf (lactic acid, glycerol, glycine, succinic acid, threonic acid), while others required larger canopy irradiation (lysine, glyceric acid, maleic acid, myoinositol, cinnamic/transferulic acid, trans-caffeic acid, and raffinose, Figure 6). Each of these metabolites is a potential signal molecule synthesized in exposed leaves and translocated to shielded organs; alternatively, an unknown signal could be transmitted to shielded tissues to induce synthesis of these compounds. Myoinositol is of particular interest in light of the microarray results. Transcripts for myo-inositol-1-phosphate synthase are down-regulated by UV-B in both irradiated and shielded leaves (Tables S3 and S4 in Additional file 2). Elevated levels of myoinositol could be a signal for down-regulation of myo-inositol-1-phosphate synthase; thus, either lowered levels of myo-inositol-1-phosphate or elevated myoinositol could be signaling molecules that coordinate UV-B responses. Other metabolites showing changes in both irradiated and shielded leaves are intermediates of primary metabolism; we hypothesize that these are unlikely to be specific signals, instead reflecting global metabolic changes induced by UV-B.

Because a signaling metabolite(s) must increase quickly in irradiated leaves to trigger transcriptome changes in shielded organs within an hour, we predicted that such molecules would show 1) high concentrations in treated leaves relative to untreated plants and 2) increases in shielded organs. In the first of 2 protocols to test these criteria, one adult leaf per plant was covered with a PE filter; a second leaf was covered with CA (allows UV-B transmittance) as a control for differences in temperature and humidity inside the sheath. After 1 and 6 h of UV-B-irradiation, leaf metabolites were compared; the PE-covered leaf should respond to UV-B only if there is a signal transmitted from exposed leaves. We also compared metabolites from PE-covered leaves in plants exposed to UV-B to those from PE-covered leaves in unirradiated plants; only the PE-covered leaf on an irradiated plant should exhibit transcript changes. Metabolites were also analyzed after WPI for 1 and 6 h and in control plants without UV-B. Figure S6 in Additional file 7 shows 27 metabolites changed by UV-B in at least one comparison. At 1 h, 26 of these show altered levels versus control plants; 9 are only changed in irradiated leaves (e.g. phosphate and fructose), while 17 are also changed in shielded leaves (e.g. shikimic, trans-caffeic, malic) (Additional file 7). Thus, metabolite changes do occur with 1 h UV-B and two-thirds of these candidate signal molecules are also detected in shielded tissues. Of the 26 metabolites that are specifically induced by UV-B, 8 show no difference to controls at 6 h whereas 11 are still altered in shielded leaves (Additional file 7).

**Figure 7:**
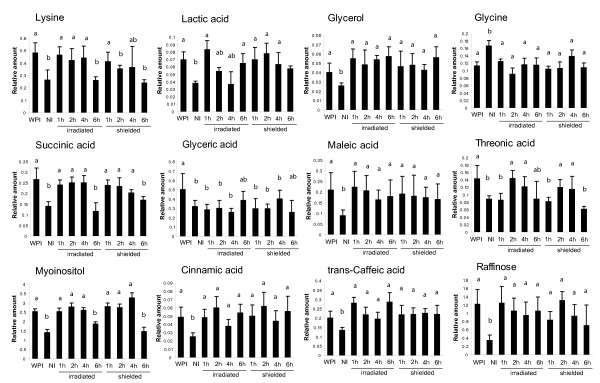
**Metabolic profiling of irradiated and shielded leaves over a time course**. Metabolic profiling of irradiated and shielded leaves from plants with two irradiated leaves over a 1, 2, 4, and 6 h time course. For statistical analysis see Materials and methods.

To analyze dynamic metabolite changes, the second protocol tested samples from two-leaf CLI at 1, 2, 4 or 6 h (Figure 1). The persistence of elevated levels (relative to non-irradiated control leaves) could be charted at a finer scale as summarized in Figure 7. Of the 12 metabolites changed at 4 h in shielded leaves (Figure 6), nearly all also show differences in shielded leaves after 1 h (Figure 7 and Figure S7 Additional file 8). Exceptions are threonic and glyceric acids which are altered after 2 and 6 h, respectively. Threonic acid is changed in irradiated and shielded leaves after only 1 h in the first protocol (Additional file 7), indicating a different threshold of canopy exposure; glyceric acid could be a secondary signal in continuing UV-B exposure. Of the 17 potential mobile signaling metabolites identified in a 1 h exposure in the first experiment, 5 were also identified after 1 h in the second protocol in both exposed and shielded leaves (lactic, succinic, trans-caffeic and maleic acids, glycerol); thus, different experiments identify the same metabolites.

The two complementary protocols generated a list of metabolites changed by UV-B in irradiated leaves and either translocated to shielded leaves or elevated there by an unknown signal. One or more of these may act as signaling compounds in shielded organ responses to UV-B in maize.

### Proteomic analysis of UV-B-irradiated and shielded leaves

**Figure 8:**
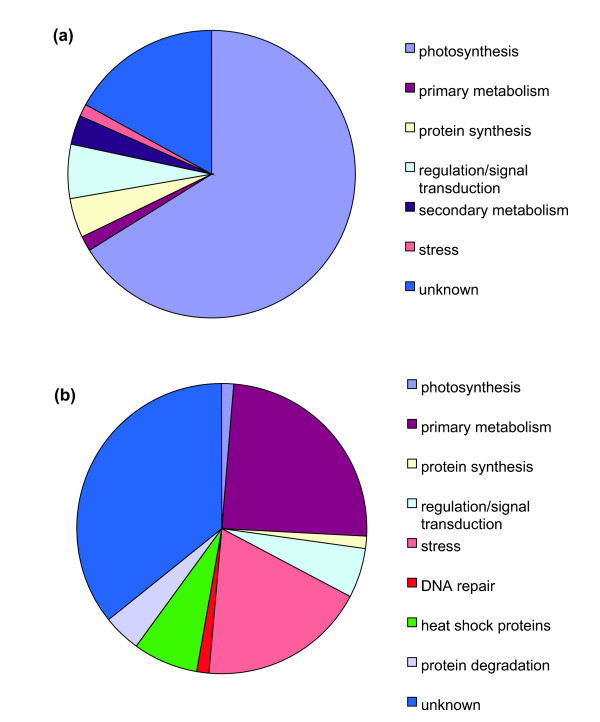
**GO Classification of UV-B-regulated proteins**. GO Classification of (a) 65 UV-B-regulated proteins in leaves from WPI or CLI samples and (b) 70 UV-B-regulated proteins in immature ears from either WPI or CLI plants. Classification is based on putative functions. For statistical analysis see Materials and methods.

To complement the above analyses, we compared changes in the leaf proteome after 2-D electrophoresis using samples from WPI, non-irradiated plants, and irradiated and shielded leaves from plants with varying CLI. Using ImageMaster 2D Platinum software, protein spots changed more than 50% (p < 0.05) in WPI plants were selected, and interpretable MS/MS spectra were obtained for 65 of them (Table S9 in Additional file 2); 29 showed increased and 36 decreased levels after WPI. For 2 and 3 irradiated leaves, 59 of the 65 identified WPI protein spots were changed by UV-B, with 33 increased and 26 decreased proteins in each case, confirming that partial canopy exposure suffices to induce most responses. Only 34 changes were confirmed with one irradiated leaf, showing that there is a graded response to exposure as was found in the microarray analysis. Plant processes highlighted by differential protein accumulation of the 65 WPI proteins are summarized in Figure 8a; these include photosynthetic proteins (66%), transcriptional regulators and proteins in signal transduction (6%), protein synthesis (4.6%), secondary metabolism (3%), and other functions (3%) while 17% of the proteins have an unknown role (Figure 8a). Many proteins were identified in several spots; these cases occur with gene family members that are differentially regulated or when one gene product is post-translationally modified. Previously, we identified a phosphorylated form of pyruvate phosphate dikinase after WPI [20]; this protein was also identified in our current analysis in different spots in both irradiated and shielded leaf samples so it is probable that this modification occurs during CLI (Table S9 in Additional file 2).

Only a subset of the changes monitored in 4 h WPI leaves were also detected in the CLI shielded leaves: 68% of the protein changes were measured in shielded leaves with 3 leaves irradiated, 58% with 2 leaves, and only 3% with a single leaf (Table S9 in Additional file 2). One kinase-like protein is increased by UV-B in shielded leaves from CLI plants; this candidate protein for UV-B signaling (Table S9 in Additional file 2) is sensitive to even low UV-B doses.

To analyze the kinetics of proteome responses to UV-B, the two leaf CLI samples from the 1, 2 or 6 h time course of irradiated and shielded leaves were analyzed along with the data from the 4 h exposure. After only 1 h of UV-B, 18 protein spots are increased and 42 are decreased by 1.5-fold (p < 0.05) in irradiated leaves; this corresponds to 92% of the total proteins changed after 4 h of UV-B exposure. Ten of the increased spots and 43 of the decreased spots are also changed in shielded leaves (Table S9 in Additional file 2). With most protein changes occurring within 1 h, it is likely that many correspond to post-translational modifications, or, in the case of decreases, protein degradation. For example, most of the decreased proteins correspond to photosynthetic proteins, particularly photosystem II components; degradation of these proteins after UV-B has been previously reported [21,22]. Many protein changes persist over the duration tested; 52 of the 65 proteins changed after 4 h of UV-B are detectably altered at 6 h (Table S9 in Additional file 2).

### Proteomic analysis of immature ears from UV-B-irradiated maize plants

In ears, 11 protein spots showed increased and 59 showed decreased levels after UV-B-irradiation (Table S10 in Additional file 2); most alterations occurred after both WPI and CLI treatments (Table S10 in Additional file 2), indicating that even modest UV-B exposure triggers proteomic changes in this reproductive organ. Plant processes highlighted by differential protein accumulation are summarized in Figure 8b; classes include enzymes of primary metabolism (24%), proteins in stress responses (18.6%), HSPs (7%), transcriptional regulation and signal transduction (5.7%) and other functions (4.2%); 35% correspond to proteins with unknown function. When analyzing the time course of responses, 73% of the protein changes measured occurred within 1 h of UV-B, while 76% of the total remains changed after 6 h (Table S10 in Additional file 2). As seen with shielded leaves, most protein changes occur quickly and a subset persist throughout longer exposures. It is interesting to note that as shown in Figures 8a and 8b, proteins changed by UV-B vary by organ, similar to transcriptome profiling observations (Additional file 8). As identified by transcriptome profiling and confirmed in the proteome, many HSPs show decreased levels in immature ears (Table S10 in Additional file 2).

## Discussion

Our focus was to identify systemic responses after UV-B-irradiation that elicit physiological changes in shielded organs. We also sought to correlate transcriptome changes to alterations in the proteome and metabolome both as cross-validation and to extract a more meaningful analysis of maize responses to UV-B. The first question addressed was how many canopy leaves must receive UV-B to alter transcripts in shielded leaves and ears. Assessing total transcripts, we found that exposure of just the top leaf substantially alters transcriptomes in irradiated leaf and shielded organs with greater changes as additional leaves are irradiated. Because the magnitude of responses was similar for two or three leaves, we conclude that exposure of only 10% of leaf area in adult maize plants suffices to elicit most responses (61%) with further responses (39% of total) requiring WPI (Additional file 1).

Transcript types specific to irradiated leaves include several genes in the flavonoid pathway, and concomitantly some phenylpropanoid precursors, such as shikimic, quinic, and trans-caffeoylquinic acids are increased by UV-B only in irradiated leaves (Additional file 6). Changes in other phenylpropanoid precursors, including cinnamic and trans-caffeic acids, occur in both irradiated and shielded leaves as does the increase in an isoflavone reductase-like1 protein (Table S9 in Additional file 2). Consequently, synthesis of specific secondary metabolites appears to result from both shared and distinctive regulation in shielded and irradiated tissues.

A number of transcripts that encode genes that participate in cell wall metabolism are induced by UV-B (3-deoxy-D-arabino heptulosonate-7-phosphate synthase, hydroxycinnamoyl CoA quinate transferase, UDP-glucuronosyltransferase; Table S2 in Additional file 2); these also correlate with the changes in phenylpropanoid precursors. Although we did not analyze metabolites in immature ears in this study, it is interesting that a number of transcripts that encode enzymes in cell wall metabolism are also changed by UV-B in these organs (cinnamyl alcohol dehydrogenase, cinnamoyl CoA reductase, cellulose synthase, beta-fructofuranosidase, etc.; Table S5 in Additional file 2). Thus, changes in cell wall structure may be a general acclimation response to UV-B in maize.

Transcripts for a myoinositol-1-phosphate synthase show complex regulation during UV-B responses. After 1 and 2 h treatments, myo-inositol-1-phosphate synthase (GRMZM2G155242, Table S7 in Additional file 2) transcripts increase in both irradiated and shielded leaves, but then decrease at 4 h. Myoinositol is synthesized from glucose-6-P, which is converted to myoinositol-1-phosphate by myoinositol-1-phosphate synthase, and this compound is dephosphorylated to produce myoinositol. The step catalyzed by the synthase is rate-limiting for myoinositol biosynthesis in plants [23,24]. Myoinositol is a candidate UV-B signaling compound, because it is rapidly increased in irradiated and shielded leaves. Rapid synthesis after brief exposure is expected for a signal, while longer irradiation (4 h or longer) could provoke a down-regulation to modulate metabolite levels. Myoinositol has been proposed as important in stress protection [25], and may also regulate programmed cell death in Arabidopsis. Mutants in one myoinositol-1-phosphate synthase (*AtIPS1*) exhibit accelerated cell death [13]; the encoded protein has a nuclear localization sequence, suggesting that nuclear pools of myoinositol may be critical [13].

UVR8 is a UV-B-specific signaling component in Arabidopsis that mediates low fluence photomorphogenic responses, and it is required for UV-B induced expression of the gene encoding the HY5 transcription factor in co-operation with COP1 [8,9]. UVR8 and COP1 interact directly and rapidly in the nucleus *in planta *after UV-B exposure [26], and this very early step in UV-B signaling is proposed to initiate UV-B acclimation [14]. The action spectrum for the induction of HY5 transcripts by UVR8 has a maximum near 280 nm with significant action at longer UV-B wavelengths; UVR8 may act as a plant photoreceptor that can mediate UV-B-specific responses [27]. Although Arabidopsis UVR8 has not been reported to be UV-B-regulated, interrogating microarray data with Genevestigator^© ^shows that UVR8 is down-regulated by UV-B (data not shown), in agreement with our maize results. In contrast, after 1 h canopy exposure, shielded maize leaves show up-regulation of UVR8 (Table S7 in Additional file 2). We speculate that if UVR8 is a UV-B sensor in maize, it may be induced very quickly in irradiated leaves and then down-regulated within 1 h. Systemic signaling results in up-regulation in shielded organs after a delay, a step that may be an acclimation to increase subsequent sensitivity to UV-B

Shielded immature ears are highly responsive to canopy irradiation. Interestingly, although the classes of genes affected in shielded leaves and ears are similar, different genes are regulated. An exception is that 15 nucleic acid binding proteins, including TFs, are UV-B-regulated in a similar manner in these shielded organs (Table S6 in Additional file 2). We propose that UV-B signaling from irradiated to shielded organs elicits common regulation of some TFs to modulate similar processes via organ-specific gene family members. Other transcripts that are similarly regulated in shielded organs encode four proteins in signal transduction (Table S6 in Additional file 2), and these are candidates for processing the UV-B signaling from irradiated to shielded tissues. Moreover, both shielded leaves and immature ears show down-regulation of HSPs, a result validated by the proteomic analysis (Tables S4, S5 and S10 in Additional file 2).

In a two leaf CLI time course, there were significant transcriptome, proteome, and metabolome changes under all conditions studied in both shielded and irradiated organs. Some required only 1 h of exposure, defining likely responses in early UV-B signaling. One transcript up-regulated after 1 h is similar to Arabidopsis PFT1, a subunit of the Mediator complex. PFT1 was first described as a positive regulator of shade avoidance in *Arabidopsis thaliana*, and it was hypothesized that it could act downstream of phytochrome B to promote flowering in response to shade [28]; however, *PFT1 *is now considered to be a gene that negatively regulates the phytochrome signaling pathway [29]. In addition, PFT1 is a positive regulator of jasmonic acid signaling during fungal pathogen infection [30]. We hypothesize that PFT1 acts in early UV-B signaling in irradiated maize leaves, perhaps contributing to the dramatic and rapid reduction in transcript diversity.

Shielded leaf transcriptomes are also dramatically remodeled during UV-B responses, although to a lesser extent than in directly irradiated leaves. There are 106 responses in common, even with 1 h exposure, including a wound and phytochrome signaling involved receptor-like kinase. Transcripts are increased both in irradiated and shielded leaves after 1 h, emphasizing its potential as a general participant in UV-B signaling. In immature ears, there are fewer total transcripts changed by UV-B than in leaves. The shielded leaf blades are within 10 cm of the irradiated canopy, whereas the immature ears are ~30 cm distant. The delayed decrease in transcriptome diversity in ears most likely reflects the longer time required for signals to reach this organ. Although the focus was on early responses, after 6 h of UV-B exposure we identified a number of newly induced transcripts. For example, up-regulated transcripts correspond to enzymes in the phenylpropanoid pathway for the synthesis of flavonoids and cell wall, stress responses, TFs of the WRKY type, and PR proteins, indicating that there are continuing acclimation responses for the duration of UV-B treatments.

Our criteria for identifying candidate signaling metabolite(s) were that the compound must increase quickly in irradiated leaves relative to untreated plants and must also increase in shielded organs. After 1 h of UV-B in two irradiation protocols, 20 metabolites met these criteria (Figure 7 and Additional file 8). In the future, pharmacological and genetic knockout strategies can be used to test the relevance of these candidate molecules to UV-B systemic signaling.

Based on 2D gels, 65 and 70 protein spots were changed in leaves and immature ears, respectively. Consequently, even a small canopy exposure suffices to modulate some abundant cellular proteins; 27% of these proteins were changed when only one leaf was UV-B-irradiated. The proteomics assessment validates the microarray results in demonstrating that for those responses detected in CLI protocols, two leaves are sufficient to elicit most proteome changes. The leaf proteome after WPI shares only a subset of responses with shielded leaves in the CLI protocols after 4 h irradiation. One kinase-like protein is increased by UV-B in shielded leaves even with just one leaf exposure; this may be another candidate protein in UV-B signaling. Another key observation is that 92% of the proteins changed after 4 h in leaves and 73% in immature ears were also changed after 1 h of UV-B. Thus, most protein changes detected occur quickly. For novel spots, post-translational modification is likely; previously, we identified pyruvate phosphate dikinase to be phosphorylated after UV-B of WPI [4], and this protein was also identified in multiple spots in irradiated and shielded leaf samples. For decreased protein abundance, rapid proteolysis must be triggered by UV-B.

Most identified proteins changed by UV-B correspond to abundant proteins, such as photosynthetic proteins in leaves (Table S9 in Additional file 2), enzymes participating in general metabolism, and heat shock and 14-3-3-like proteins (Tables S9 and S10 in Additional file 2). In this group of highly abundant proteins, we found a good correlation between microarray and proteomic results. For example, in leaves, UV-B induces both transcript and protein levels of ferredoxin, pyruvate phosphate dikinase and fructose 1,6 biphosphate aldolase; while RuBisCO is decreased both at transcript and protein levels (Tables S1 and S9 in Additional file 2). A similar correlation exists for the down regulation of HSP70 at both transcript and protein levels in immature ears (Tables S5 and S10 in Additional file 2). For other genes, however, a correlation between protein and transcript level changes is only seen when different time points are compared, probably because new proteins require longer times to be produced. Examples of this temporally delayed correlation are oxygen-evolving enhancer protein, a chlorophyll a-b binding protein in leaves (Tables S1, S7, and S9 in Additional file 2), and an inorganic phosphatase (Tables S5 and S10 in Additional file 2). Finally, a few proteins showed opposite UV-B regulation compared to transcript changes; this is the case in leaves for 50S ribosomal protein L12 (down regulation at the transcript level, up regulation at the protein level) and glycosyl transferase family 8 (up regulation at the transcript level, down regulation at the protein level; Tables S1 and S9 in Additional file 2). With respect to metabolite changes, it is important to note that changes measured at the transcript and proteome level of photosynthetic proteins and enzymes in primary metabolism, are reflected in changes in the total pools of soluble metabolites. For example, pools of molecules that are intermediates or products of glycolysis, the Krebs cycle and fermentation pathways (lactic acid, succinic acid, glycerate, 2-ketoglucaric acid, etc.) are changed by UV-B as a consequence of metabolic pathway fluxes (Additional files 6, 7 and 8). In addition, soluble sugars, products of photosynthesis are also significantly changed after the UV-B treatments (glucose, fructose, galactose, etc.; Additional files 6, 7 and 8).

## Conclusions

**Figure 9:**
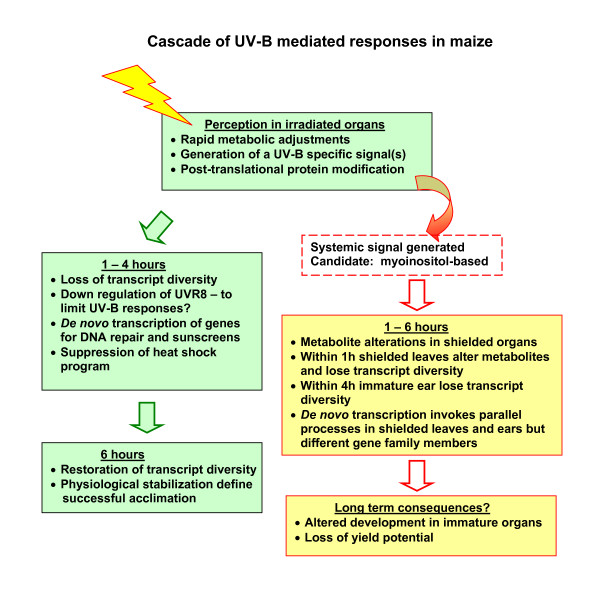
**Schematic summary of key events of maize responses to UV-B**.

Our focus was to document the scope and kinetics of systemic changes in shielded leaves and immature ears after irradiation of canopy leaves and to compare irradiated and shielded organs over a time course. Transcriptome and proteome profiling was used to track macromolecular alterations in exposed and shielded organs. In parallel, metabolome profiling was used to search for candidate signaling molecules. Figure 9 shows a summary of the results obtained and outlines major conclusions. We showed that direct exposure of just the top leaf substantially alters the transcriptome of both irradiated and shielded organs, with greater changes as additional leaves are irradiated. Some phenylpropanoid pathway genes are expressed only in irradiated leaves, reflected by the accumulation of some phenylpropanoid precursors only in these leaves. Transcriptome, proteome, and metabolome changes are also UV-B-regulated in shielded organs; shielded leaf transcriptomes are dramatically remodeled during UV-B responses. After 1 h of UV-B in two different protocols, 20 metabolites increased quickly in irradiated leaves relative to untreated plants and also increased in shielded organs. Thus, synthesis of specific secondary metabolites appears to result from both shared and distinctive regulation in shielded and irradiated tissues. Candidates for components of signal transduction and possible signal molecules were identified utilizing a time course experiment; for example, myoinositol or a derivative is a signaling candidate(s) in UV-B responses (Figure 9). Collectively, the results presented here highlight possible signaling pathways and molecules for future research. An important next step is understanding the regulatory networks that permit acclimation responses to UV-B.

## Methods

### Samples and Treatments

W23 maize was grown for 5 weeks in the greenhouse during July and August 2008 using the same protocol as described previously [6]. The afternoon prior to treatment, 12 plants were moved underneath UV lamps, and the topmost leaves (either 1, 2 or 3) were threaded through slits in PE plastic; after acclimating overnight, these leaves received 1, 2, 4, or 6 h UV-B exposure [6]. Exposure times were centered on 11 am, 5h after sunrise and supplemental greenhouse lighting (33% of summer noon solar fluence). Two different controls were utilized: an untreated (no irradiation treatment, NI) and whole plant irradiation (WPI); these plants were moved into the irradiation apparatus as described above to minimize detection of any gene expression response resulting from moving the plants into position in the grid the afternoon prior. The four hour, 2-leaf irradiated treatment was also included as a linking control between the two types of experiments. A biological replicate consisted of tissue samples pooled from 4 plants. Four biological replicates for each treatment (a total of 16 plants) were used in all assays, except for the WPI and 6 h treatment, where only two biological replicates were used in the microarray experiments (Figure 1a). The three sample types (irradiated leaf, shielded leaf, and immature ear) were immediately flash-frozen in liquid nitrogen after harvesting. Irradiated leaf CLI samples were collected from each irradiated leaf while WPI leaf samples were taken from the top 2 canopy leaves. Shielded leaf samples were taken from the 2 leaves immediately below the PE plastic. Because of space limitations in the UV-B irradiation apparatus (Figure 1b), only 12 plants could be processed at a time. Therefore one biological replicate for each sample and treatment type was always done on a subsequent day, but always during the same time period. Leaves were harvested by one person while two researchers dissected and harvested immature ears. Irradiated leaves were cut before cutting the plants and taking them to the work area, where ears and both leaf sample types were harvested and flash frozen. Total time to harvest all samples was approximately 10 min; leaves were harvested within about 5 min and ears took about 10 min. Leaves were separated from the midrib before being frozen. Immature ears (1-6 cm) were cut in half and distributed randomly among the different collection tubes for RNA or proteomics analysis.

### Microarray experiments

RNA extraction and microarray hybridization were done as described in Casati and Walbot [31]. Data acquisition, image processing, and spot flagging and removal were performed as described in Skibbe *et al *[32]. The median foreground values for each channel from the Agilent Feature Extraction software were first normalized using the lowess method of the limma package in R [33] (within each array) and then using limma's quantile method (between all arrays). Probes were classified as "on" if their expression value was more than 3.0 standard deviations above the average foreground intensity of the Agilent negative controls, providing a 0.13% FDR (~ 50 probes). For differential expression, we used the unadjusted p-value generated by limma for FDR. Differentially expressed probes were identified using either a 1.5 or 2 fold cutoff for expression ratios with a limma-assigned p-value < 0.05. Probes were included in the analysis if at least 75% of the replicate expression values (*i.e*. 6 of 8, 3 of 4, or 2 of 2) were classified as "on". Microarray data were deposited in GEO under ID GSE25038.

### Protein extraction and proteomic analysis

Protein extraction, labeling, 2D gel electrophoresis, gel image analysis, MS, and database search were done as described in Falcone-Ferreyra *et al*. [34].

### Metabolite profiling

Extraction, liquid partition, and derivation prior to GC-MS analysis were performed as described by Lisec *et al*. [35]. For analysis, four biological replicates per treatment with a second group of technical replicates were utilized (8 total data points used during analysis). GC-MS analysis was performed using an autosystem XL Gas Chromatograph and a Turbo Mass Spectrometer (Perkin Elmer) in the Facultad de Ciencias Bioquímicas y Farmacéuticas - UNR facilities. One μL split injection (split ratio 1:40) was injected at 280°C. The capillary column used was aVF-5ms column (Varian, Darmstadt, Germany) with the following dimensions: 30 m × 0.25 mm inner diameter and a 0.25 μm film with helium as carrier gas with constant flow at 1 mL/min. The temperature program was 5 min at 70ºC, 5 min ramp to 310ºC and final heating for 2 min at 310ºC. The transfer line to the MS was set to 280ºC. Spectra were monitored in the mass range *m*/*z *= 70-600. Tuning and all other settings were according to manufacturer's recommendations.

Chromatograms were acquired with TurboMass 4.1 software (Perkin Elmer). The NIST98mass spectral search program (http://www.nist.gov/srd/mslist.htm, National Institute of Standards and Technology, Gaithersburg, MD, USA) was the software platform. The MS and retention time index were compared with the collection of the Golm Metabolome Database [36,37]. MS matching was manually supervised and matches accepted with thresholds of match > 650 (with maximum match equal to 1000) and retention index deviation < 1.0%. Peak heights were normalized using the amount of the sample fresh weight and ribitol for internal standardization. Relative metabolite contents were determined and statistical analyses were performed using ANOVA tests in Sigma Stat 3.1.

## Abbreviations

UV-B: ultraviolet-B; WPI: whole plant irradiation; TF: transcription factor; CLI: Canopy leaf irradiation; NI: No irradiation; CA: cellulose acetate; PE: polyester; HSP: heat shock proteins; PFT1: phytochrome and flowering time 1 protein; GST: gluthatione-S-transferase; MS/MS: tandem mass spectrometry.

## Competing interests

The authors declare that they have no competing interests.

## Authors' contributions

PC and VW conceived the experiments, VW and DJM grew the plants and carried out the UV-B experiments, DJM did RNA, protein and metabolite extractions, DJM did the RNA labeling and microarray hybridizations, JF and PC analyzed the microarray experiments, MC did the proteomic experiments, PC did the metabolomic analysis. The paper was written by PC, VW, and DJM and edited by MC and JF. All authors have read and approved the final manuscript.

## Supplementary Material

Additional file 1 **Figure S1**. Classification of UV-B-regulated genes identified by microarrays based on their putative function in fully UV-B-irradiated plants (WPI). (a and b) transcripts that are up (a) and down (b) regulated in fully UV-B-irradiated plants; (c and d) transcripts that are up (c) and down (d) regulated only in fully UV-B-irradiated plants and not when plants are only irradiated in 1, 2 or 3 leaves per plant. Classification was done for the UV-B-regulated transcripts that are changed at least 2-fold (p < 0.05). Click here for file

Additional file 2 **Supplemental Tables**. Click here for file

Additional file 3 **Figure S2**. (a) Venn diagrams comparing transcriptome changes in shielded leaves that were irradiated in the absence of UV-B. Up-regulated genes are in red, down-regulated genes are in green. Click here for file

Additional file 4 **Figure S3**. Venn diagrams comparing transcriptome changes in leaves that were covered with a plastic sheath that absorbs UV-B. Only two adult leaves per plant were irradiated over a time course of 1, 2, 4, and 6 h. Up-regulated genes are in red, down-regulated genes are in green. (a) Intersection of genes differentially expressed in irradiated leaves; (b) Intersection of genes differentially expressed in shielded leaves; (c) Intersection of genes differentially expressed in immature ears. Each sample was compared to plants under control conditions in the absence of UV-B (NI). Transcripts showing changes higher than 2-fold (p < 0.05) were included in the classification. Click here for file

Additional file 5 **Figure S4**. GO classification of transcripts into categories: those that were turned on (OnOff), or off (OffOn), or that were up- or down-regulated over the 6 h time course experiment were used. Transcripts that belonged to fifteen major cellular processes were used for the classification. Click here for file

Additional file 6 **Figure S5**. Metabolic profiling of irradiated and shielded leaves from fully UV-B-irradiated leaves for 4 h (WPI), and control untreated leaves (NI) are included. All metabolites that are changed by UV-B are in red, while down-regulated transcripts by 2-fold are in green. Click here for file

Additional file 7 **Figure S6**. Metabolic profiling of irradiated and 6 h in 2 leaves with control untreated plants during 1 and 6 h. As a control, samples from fully irradiated leaves for 4 h (UV-B), and control untreated leaves (NI) are included. CA/PE: comparison of metabolite levels in leaves covered with a plastic that allows UV-B transmittance (CA) vs. levels in leaves covered with a plastic sheath that absorbs UV-B (PE, see Material and methods); PE UV-B/C: comparison of metabolites from PE-covered leaves in plants exposed to UV-B to those from PE-covered leaves in non-irradiated plants; UV-B/CA: metabolite level comparison in leaves that are directly UV-B-irradiated vs. levels in leaves covered with a plastic that allows UV-B transmittance (CA). Statistical analysis was done using one way ANOVA; statistically significant differences are labeled with * (α = 0.05). Click here for file

Additional file 8 **Figure S7**. Metabolic profiling of irradiated and shielded leaves with varying canopy exposure to UV-B radiation. As a control, samples from fully UV-B-irradiated leaves for 4 h (UV-B), and control untreated leaves (C) are included. Statistical analysis was done using one way ANOVA; statistically significant differences are labeled with letters a and b (α = 0.05). Click here for file
