# Supplementary material for: Transcriptomic, proteomic and metabolomic analysis of UV-B signaling in maize
Source: BMC Genomics. 2011 Jun 16;12:321. doi: 10.1186/1471-2164-12-321 (PMC3141669; doi:10.1186/1471-2164-12-321)

Transcripts that are up regulated by UV-B in fully exposed plants (2-fold, total 203)

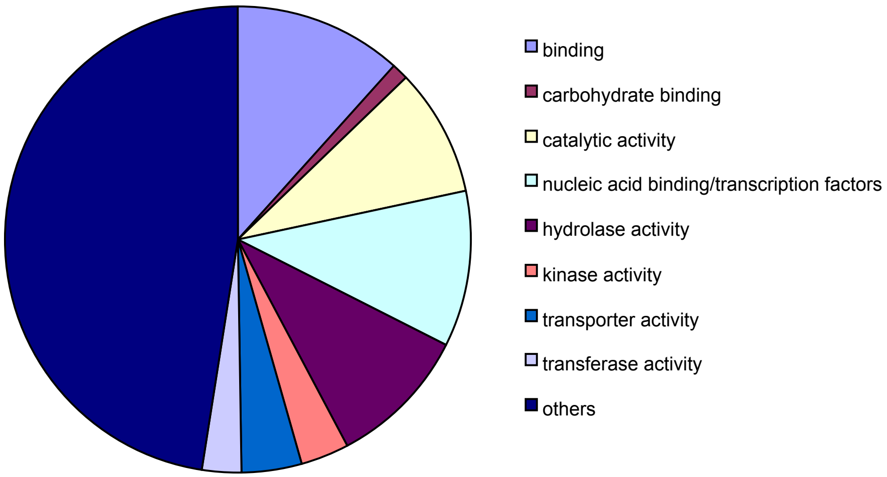

Transcripts that are down regulated by UV-B in fully exposed plants (2-fold, total 213)

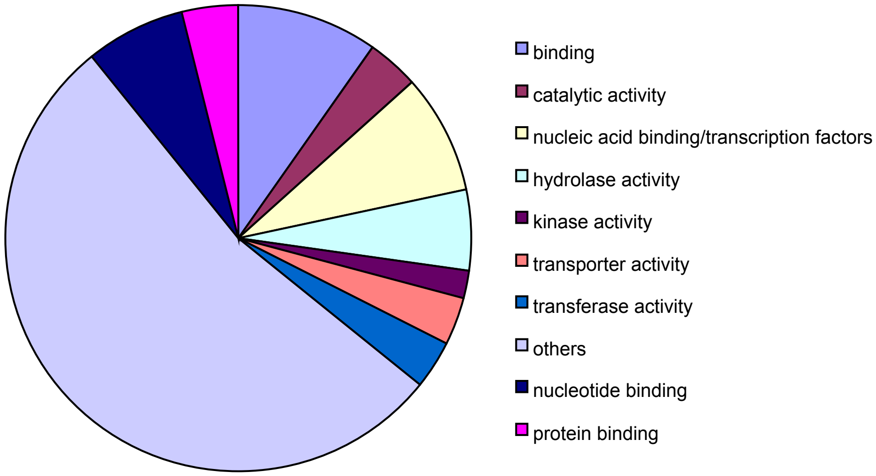

Transcripts that are only up regulated by UV-B in fully exposed plants (2-fold, total 82)

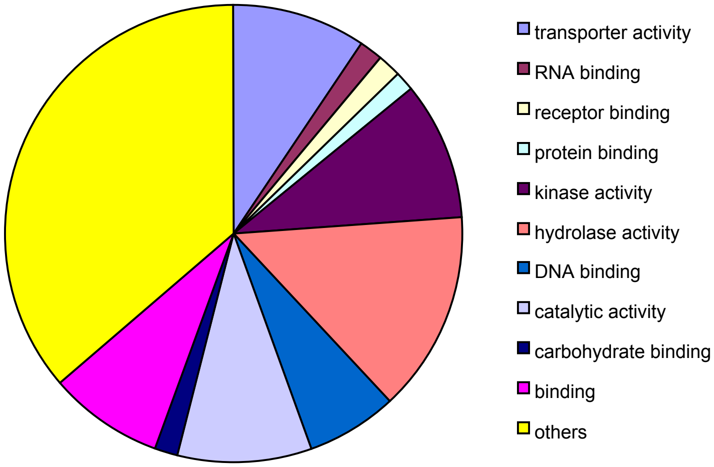

Transcripts that are only down regulated by UV-B in fully exposed plants (2-fold, total 81)

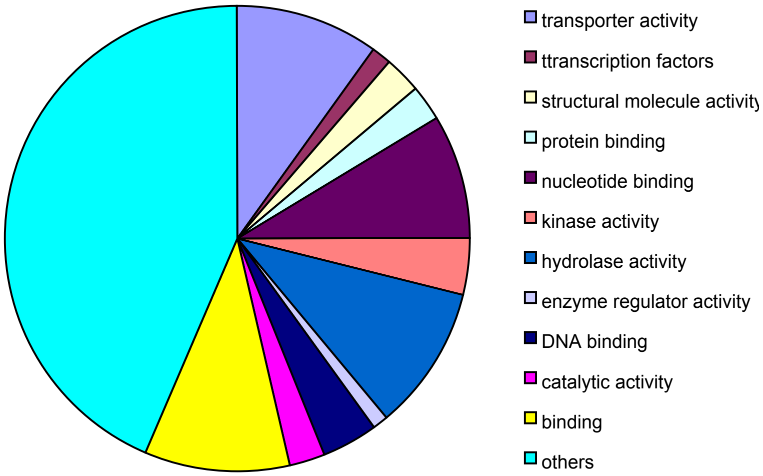

Supplement: Additional file 1 — Figure S1. Classification of UV-B-regulated genes identified by microarrays based on their putative function in fully UV-B-irradiated plants (WPI). (a and b) transcripts that are up (a) and down (b) regulated in fully UV-B-irradiated plants; (c and d) transcripts that are up (c) and down (d) regulated only in fully UV-B-irradiated plants and not when plants are only irradiated in 1, 2 or 3 leaves per plant. Classification was done for the UV-B-regulated transcripts that are changed at least 2-fold (p < 0.05). [file 1471-2164-12-321-S1.PDF]
