# Supplementary figures and images for: Transcriptomic, proteomic and metabolomic analysis of UV-B signaling in maize
Source: BMC Genomics. 2011 Jun 16;12:321. doi: 10.1186/1471-2164-12-321 (PMC3141669; doi:10.1186/1471-2164-12-321)

(a)

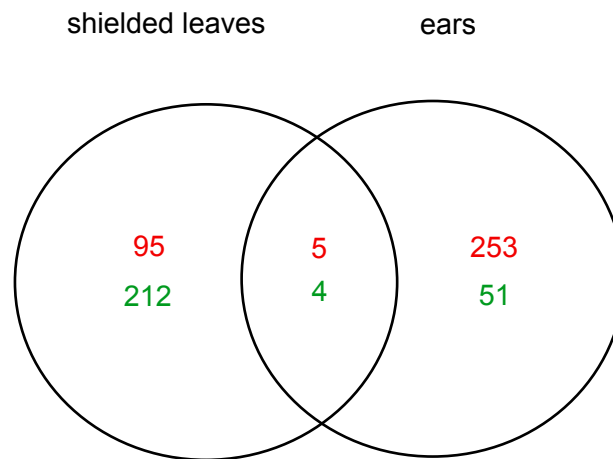

(b)

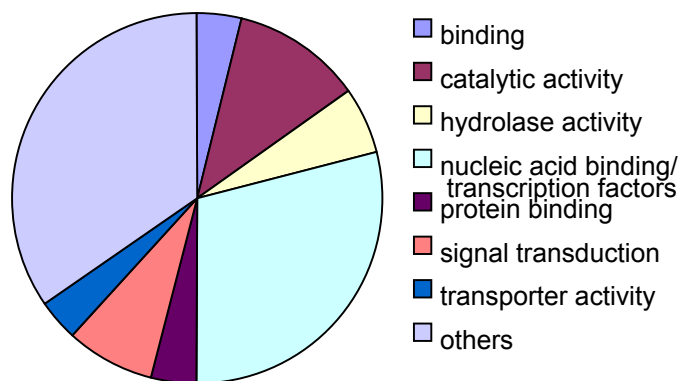

Supplement: Additional file 3 — Figure S2. (a) Venn diagrams comparing transcriptome changes in shielded leaves that were irradiated in the absence of UV-B. Up-regulated genes are in red, down-regulated genes are in green. [file 1471-2164-12-321-S3.PDF]

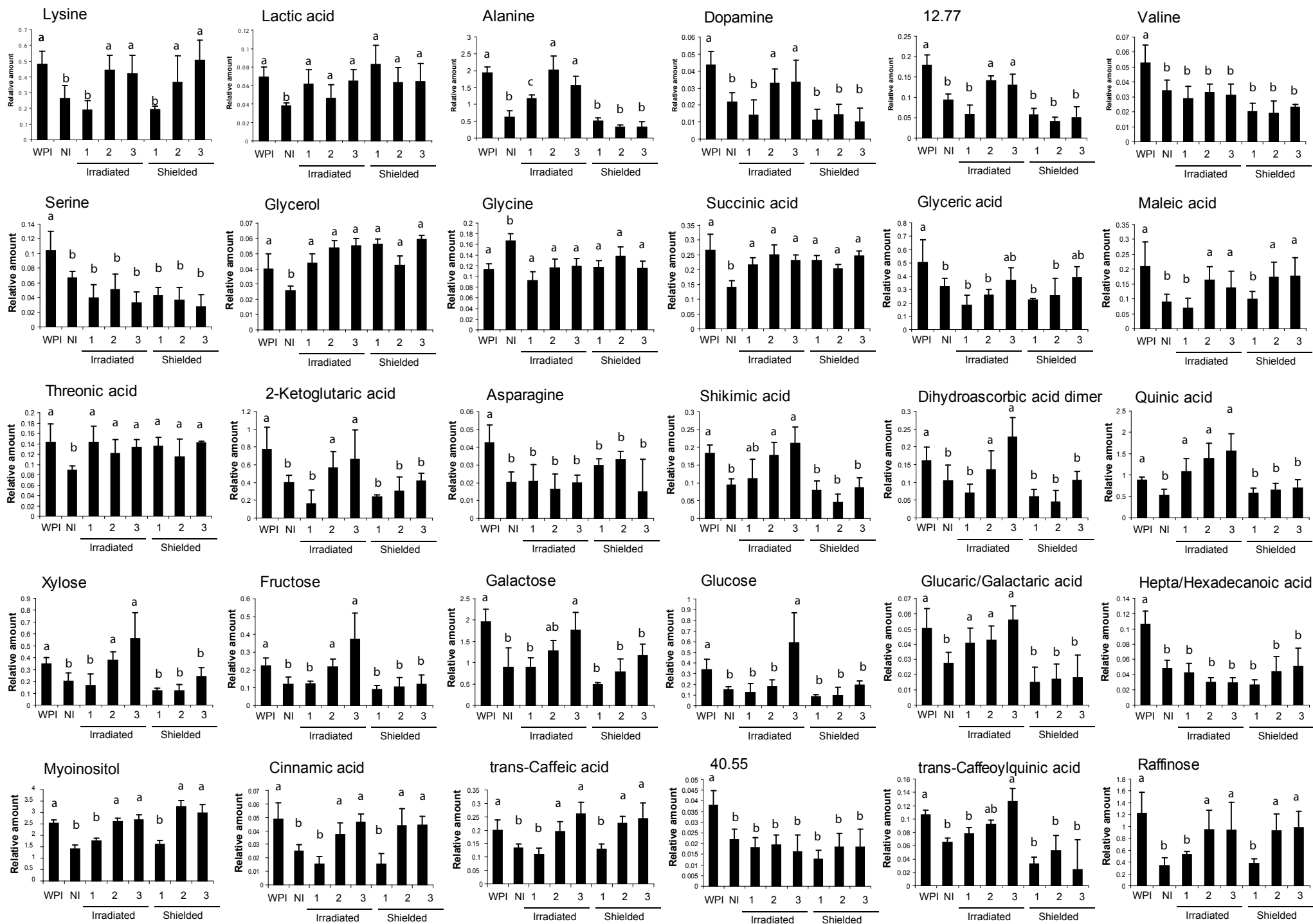

Supplement: Additional file 6 — Figure S5. Metabolic profiling of irradiated and shielded leaves from fully UV-B-irradiated leaves for 4 h (WPI), and control untreated leaves (NI) are included. All metabolites that are changed by UV-B are in red, while down-regulated transcripts by 2-fold are in green. [file 1471-2164-12-321-S6.PDF]

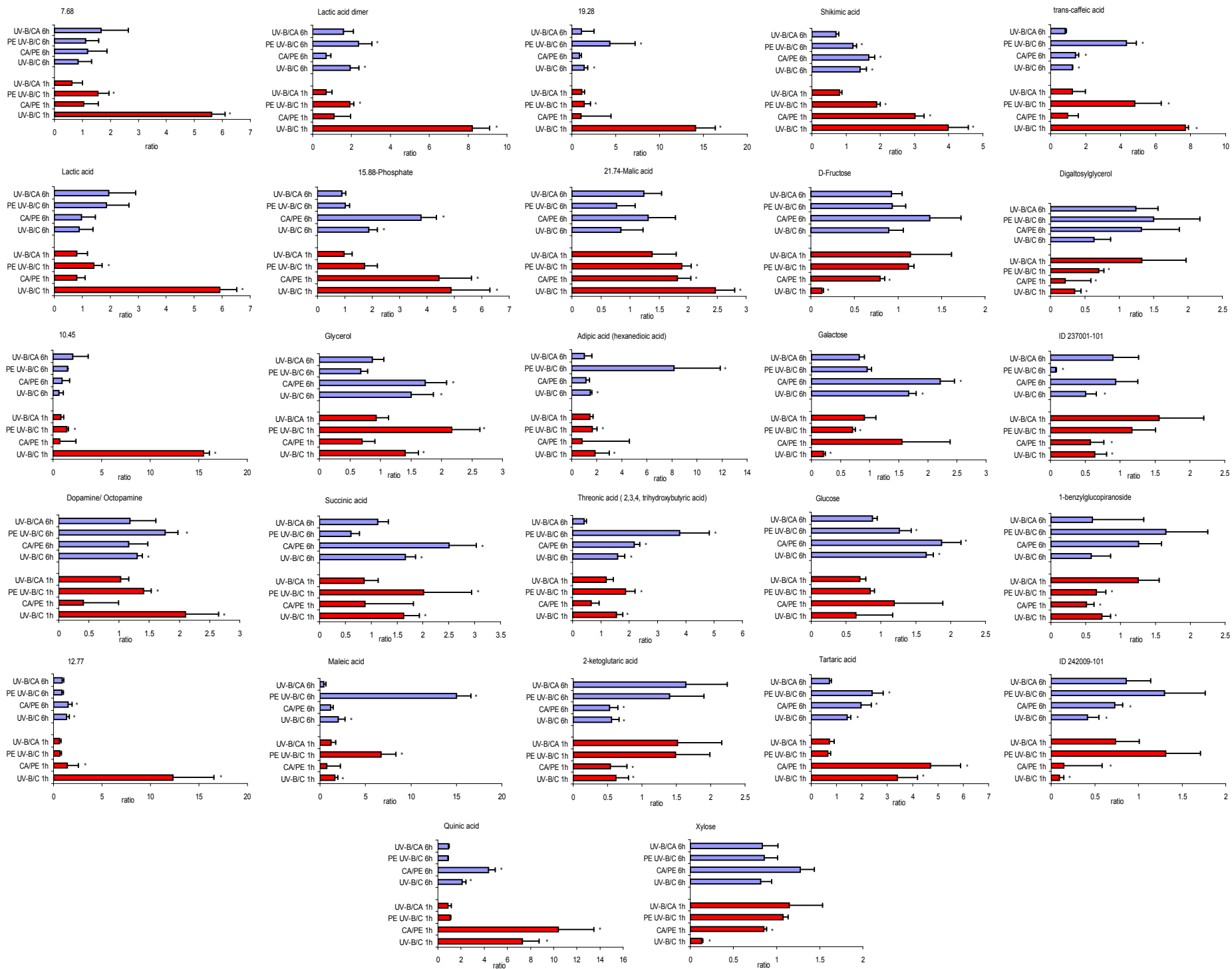

Supplement: Additional file 7 — Figure S6. Metabolic profiling of irradiated and 6 h in 2 leaves with control untreated plants during 1 and 6 h. As a control, samples from fully irradiated leaves for 4 h (UV-B), and control untreated leaves (NI) are included. CA/PE: comparison of metabolite levels in leaves covered with a plastic that allows UV-B transmittance (CA) vs. levels in leaves covered with a plastic sheath that absorbs UV-B (PE, see Material and methods); PE UV-B/C: comparison of metabolites from PE-covered leaves in plants exposed to UV-B to those from PE-covered leaves in non-irradiated plants; UV-B/CA: metabolite level comparison in leaves that are directly UV-B-irradiated vs. levels in leaves covered with a plastic that allows UV-B transmittance (CA). Statistical analysis was done using one way ANOVA; statistically significant differences are labeled with * (α = 0.05). [file 1471-2164-12-321-S7.PDF]

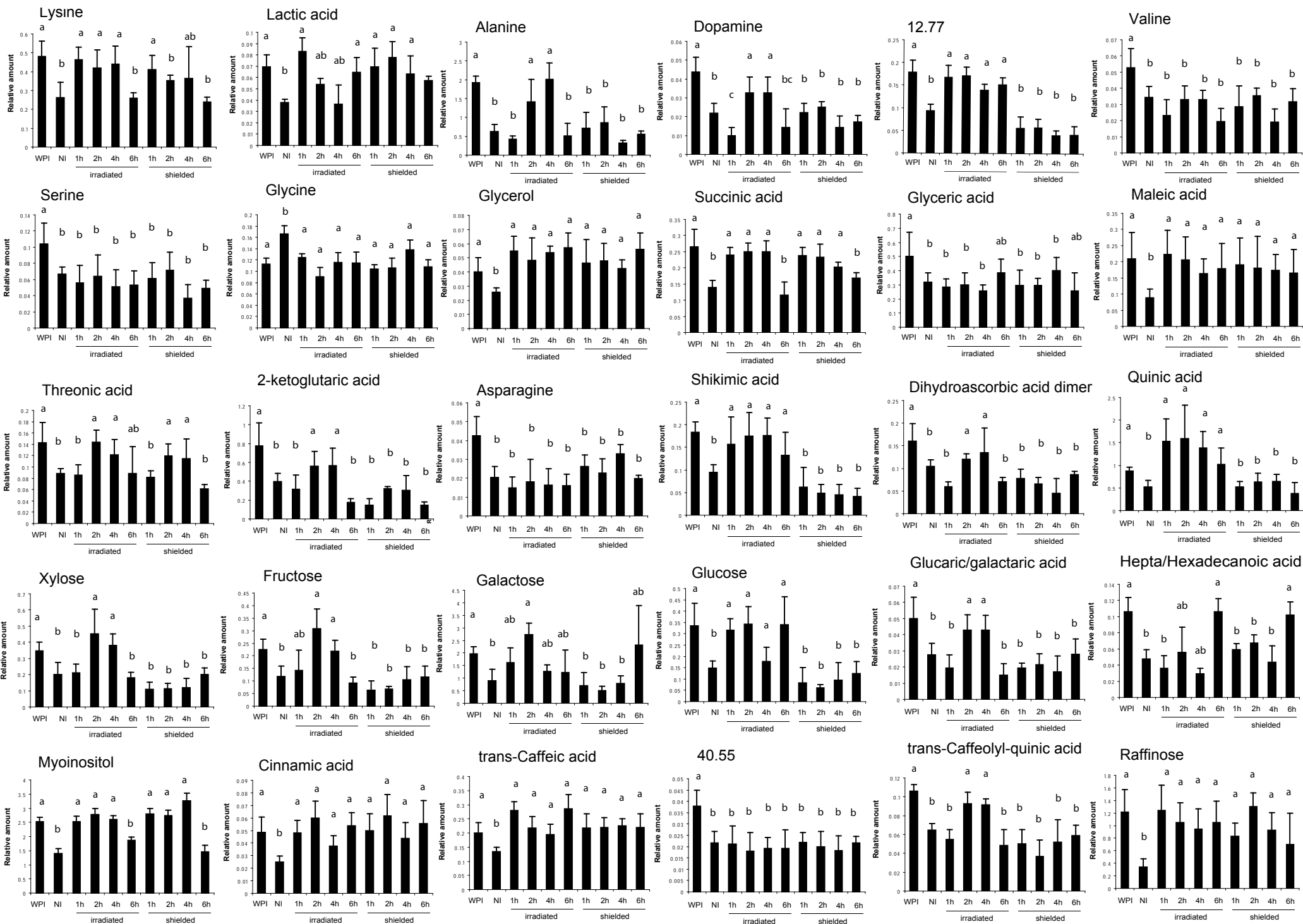

Supplement: Additional file 8 — Figure S7. Metabolic profiling of irradiated and shielded leaves with varying canopy exposure to UV-B radiation. As a control, samples from fully UV-B-irradiated leaves for 4 h (UV-B), and control untreated leaves (C) are included. Statistical analysis was done using one way ANOVA; statistically significant differences are labeled with letters a and b (α = 0.05). [file 1471-2164-12-321-S8.PDF]
