# Supplementary material for: Transcriptomic, proteomic and metabolomic analysis of UV-B signaling in maize
Source: BMC Genomics. 2011 Jun 16;12:321. doi: 10.1186/1471-2164-12-321 (PMC3141669; doi:10.1186/1471-2164-12-321)
Supplement: Additional file 4 — Figure S3. Venn diagrams comparing transcriptome changes in leaves that were covered with a plastic sheath that absorbs UV-B. Only two adult leaves per plant were irradiated over a time course of 1, 2, 4, and 6 h. Up-regulated genes are in red, down-regulated genes are in green. (a) Intersection of genes differentially expressed in irradiated leaves; (b) Intersection of genes differentially expressed in shielded leaves; (c) Intersection of genes differentially expressed in immature ears. Each sample was compared to plants under control conditions in the absence of UV-B (NI). Transcripts showing changes higher than 2-fold (p < 0.05) were included in the classification. [file 1471-2164-12-321-S4.PDF]

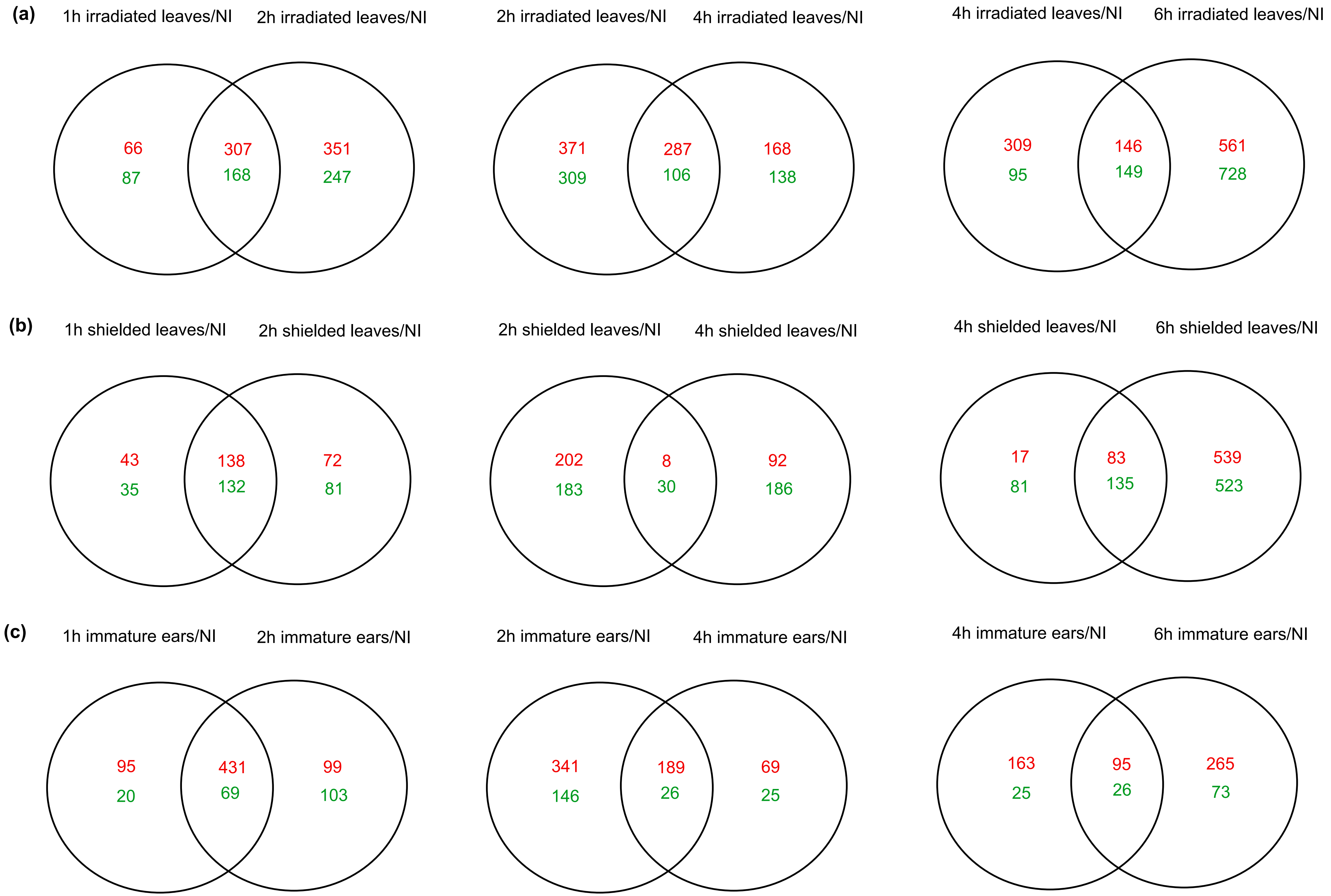
(a)
1h irradiated leaves/NI
2h irradiated leaves/NI
66
87
307
168
351
247
2h irradiated leaves/NI
4h irradiated leaves/NI
371
309
287
106
168
138
4h irradiated leaves/NI
6h irradiated leaves/NI
309
95
146
149
561
728
(b)
1h shielded leaves/NI
2h shielded leaves/NI
43
35
138
132
72
81
2h shielded leaves/NI
4h shielded leaves/NI
202
183
8
30
92
186
4h shielded leaves/NI
6h shielded leaves/NI
17
81
83
135
539
523
(c)
1h immature ears/NI
2h immature ears/NI
95
20
431
69
99
103
2h immature ears/NI
4h immature ears/NI
341
146
189
26
69
25
4h immature ears/NI
6h immature ears/NI
163
25
95
26
265
73
