# Supplementary material for: Transcriptomic, proteomic and metabolomic analysis of UV-B signaling in maize
Source: BMC Genomics. 2011 Jun 16;12:321. doi: 10.1186/1471-2164-12-321 (PMC3141669; doi:10.1186/1471-2164-12-321)
Supplement: Additional file 5 — Figure S4. GO classification of transcripts into categories: those that were turned on (OnOff), or off (OffOn), or that were up- or down-regulated over the 6 h time course experiment were used. Transcripts that belonged to fifteen major cellular processes were used for the classification. [file 1471-2164-12-321-S5.DOC]

Figure S4. Probes from each time point in the time course experiment that were classified into 14 GO biological processes are shown if they fall into one of 4 expression types: turned on (OnOff) or turned off (Off On) or up- or down-regulated versus the NI control.
